# Is cognitive impairment associated with the presence and severity of peripheral neuropathy in patients with type 2 diabetes mellitus?

**DOI:** 10.1186/s13098-015-0045-0

**Published:** 2015-06-07

**Authors:** Rodrigo O. Moreira, Ana Luiza Soldera, Bruno Cury, Carolina Meireles, Rosane Kupfer

**Affiliations:** Instituto Estadual de Diabetes e Endocrinologia (IEDE), Pontificia Universidade Católica do Rio de Janeiro (PUC-RJ), Rua Moncorvo Filho 90, CEP 20211-340 Rio de Janeiro, RJ Brazil

**Keywords:** Diabetic neuropathy, Cognition, Alzheimer’s disease

## Abstract

**Background:**

Peripheral Diabetic Neuropathy (PDN) and cognitive impairment are complications of Diabetes Mellitus (DM) that seem to share several underlying mechanisms. The aim of this study was to investigate whether diabetic patients would have worse cognitive function than non diabetic individuals and within diabetic patients, whether those with PDN would present an even more significant cognitive impairment.

**Findings:**

Ninety four (94) outpatients with Type 2 DM were sequentially evaluated. Also, Fifty four (54) healthy individuals were sequentially selected to match the diabetic group. For the assessment of neuropathy, Portuguese versions of the Neuropathy Disability Score (NDS) and Neuropathy Symptom Score (NSS) were used. Global cognitive function was assessed by using the Portuguese Version of the Mini-Mental State Examination (MMSE), Trail Making Tests A and B and Verbal Fluency Test. Significantly lower scores were found in the Type 2 DM group in comparison to control group in the MMSE (25.7 [16–30] vs 27.6 [19–30]; *p* <0.001). Within T2DM group, forty five (45) patients were diagnosed with PDN. No differences were found between patients with and without PDN in all cognitive tests (*p* >0.05 in all comparison). No correlation was also found among NSS, NDS and any of the cognitive tests.

**Conclusion:**

Although diabetic patients do have a worse cognitive function, this impairment does not seem to be related to the presence and/or severity of PDN.

## Introduction

Patients with poorly controlled Type 2 Diabetes Mellitus (T2DM) usually develop complications, particularly microvascular. Among these, Diabetic Neuropathy (DN) is characterized by progressive nerve destruction, leading to innumerous different clinical presentations, including Peripheral Diabetic Neuropathy (PDN) [1]. In the past years, cognitive impairment has also been demonstrated as a complication of T2DM [2]. Although the pathogenesis has been linked mainly to impaired insulin signaling [3], some studies have suggested that it may also share multiple pathogenic pathways with PDN, including oxidative stress, inflammation, dyslipidemia, among others [4–8]. Considering the coexistence of some of these mechanisms in patients with PDN, it would be interesting to speculate whether patients with peripheral neuropathy would also exhibit some degree of central nervous system lesion. Therefore, we hypothesized that diabetic patients would have worse cognitive function than non diabetic individuals and that within diabetic patients, those with PDN would present an even more significant cognitive impairment.

## Patients and Methods

### Study population

Ninety four (94) outpatients with T2DM were sequentially evaluated in the Instituto Estadual de Diabetes e Endocrinologia do Rio de Janeiro, a tertiary referral center, from September 2011 to August 2012.

Inclusion criteria were: age higher than 60 years old, at least 04 years of formal education, more than 2 years of T2DM diagnosis and ability to understand the procedures of the study.

Exclusion criteria included previous amputation, blindness, end stage kidney disease, end stage liver disease, previous diagnosis of dementia, use of cholinesterase inhibitors, diagnosis of major psychiatric conditions (defined by the patient according to the need of psychiatric treatment), past or present coronary artery disease (including Myocardial Infarction, coronary revascularization, stable or unstable angina), cerebrovascular disease (including stroke and transitory ischemic attack) and symptomatic peripheral vascular disease. Patients were also excluded if they had any medical diseases or neurologic disorders that could be associated with neuropathy. The protocol was approved by the Ethics Committee of the Institution and written informed consent was obtained from each patient after the procedures involved in the study were fully explained.

Fifty four (54) healthy individuals were sequentially selected to match the diabetic group by age, gender and educational level. These individuals were all employees in the hospital. A complete medical history was obtained from these individuals to confirm that they have no relevant medical condition. Inclusion and exclusion criteria were the same as for the diabetic group, except that patients should not have T2DM.

### Neuropathy assessment

All patients were carefully examined by an experienced endocrinologist and provided a detailed medical history at baseline evaluation. For the assessment of neuropathy, Portuguese versions of the Neuropathy Disability Score (NDS) and Neuropathy Symptom Score (NSS) were used. The final score was the mean value of their evaluations. NSS and NDS are two of the most common instruments used for the assessment of SDPN in clinical practice and medical research. NSS and NDS have already been adequately translated into Portuguese and are both considered to be reliable instruments [9]. Moreover, they cover both symptoms and signs of SDPN. The NDS and NSS versions used in this study were derived from the version modified by Young et al. [10]. NDS was obtained with the examination of the ankle reflex, vibration, pin-prick, and temperature sensation (cold tuning fork) in the big toe. The total maximum abnormal score on this scale was 10. A score of 3–5 was regarded as evidence of mild neuropathic signs, 6–8 as evidence of moderate signs and a score of 9–10 was regarded as evidence of severe signs of neuropathy. The NSS was based on questioning the patients about their experiences of pain or discomfort in the legs. The maximum symptom score on this scale was 9. A symptom score of 3–4 was regarded as mild symptoms, a score of 5–6 as moderate symptoms and a score of 7–9 as severe symptoms of neuropathy. The minimum acceptable criteria for a diagnosis of peripheral neuropathy were: moderate signs with or without symptoms, or mild signs with moderate symptoms. Mild signs alone or with mild symptoms were not considered to be an adequate parameter for a diagnosis of peripheral neuropathy.

### Cognition assessment

Global cognitive function was assessed by using the Portuguese Version of the Mini-Mental State Examination (MMSE) [11]. Trail Making Tests A (TMT-A), B (TMT-B) and Verbal Fluency Test (VFT - Animals) were also used to evaluate general aspects of cognition.

### Statistical analysis

Statistical analysis was performed with GraphPad InStat 3.00 (GraphPad Software, San Diego, CA, USA). Comparison between groups was performed using Mann–Whitney test and Fisher’s exact test to analyze categorical data. For non parametric variables, data are presented as median [lower limit–upper limit]. Spearman test was used for correlation analysis of nonparametric variables. The level of statistical significance was 5 %.

## Results

One hundred (100) patients with DM and 60 healthy individuals were invited to join the study. Six patients in both group refused to participate.

Initially, patients with T2DM were compared with the control group. No differences were found in age, gender or years of formal education (Table 1). When cognitive function was evaluated, significantly lower scores were found in the T2DM group in comparison to control group in the MMSE (25.7 [16–30] vs 27.6 [19–30]; *p* <0.001). No differences were found in TMT-A (67.8 seconds in T2DM [30–186] vs 74.2 in control group [25–238]; *p* = 0.24) and VFT (13.7 words in T2DM [8–20] vs 13.4 in control group [6–21]; *p* = 0.58). A trend toward significance were found in TMT-B (144.0 seconds in T2DM [59–321] vs 157.7 in control group [40–296]; *p* = 0.083).

**Table 1:** Socio-demographic characteristics of a sample of patients with type 2 diabetes mellitus in comparison to control group

	Control group	Patients with diabetes	
	(*n* = 54)	(*n* = 94)	*p*
Age (years)	65.8	66.4	0.21
	(61–84)	(60–75)	
Gender (Female)	52	87	0.48
Educational Level (years)	7.6	7.5	0.83
	(4–16)	(2–18)	
Marital Status (Married)	26	49	0.73
Income (Minimun Wage)	2	2.2	0.56
	(0.5–4.0)	(1.0–8.0)	

Within T2DM group, forty five (45) patients were diagnosed with PDN. Patients with and without PDN were compared and no differences were observed in age, gender, educational level, marital status and disease duration (data not shown). Also, no differences were found between patients with and without PDN in all cognitive tests (Table 2). No correlation was also found among NSS, NDS and any of the cognitive tests (data not shown).

**Table 2:** Comparison of cognitive function in type 2 diabetic patients according to the diagnosis of peripheral diabetic neuropathy

	Without neuropaty	With neuropaty	
	(*n* = 48)	(*n* = 45)	*p*
Mini-Mental State Examination	26.0	25.5	0.58
	(16–30)	(16–30)	
Trail Making Test-A (seconds)	75.7	72.6	0.22
	(27–142)	(25–238)	
Trail Making Test-B (seconds)	153.5	163.4	0.47
	(40–294)	(55–296)	
Verbal Fluency Test	13.8	13.0	0.23
	(8–21)	(6–19)	

## Discussion

The relationship between T2DM, cognitive impairment and Alzheimer’s Disease seems strong. Although some mechanisms linking these diseases have already been established, several points are still controversial [3, 12]. In particular, it remains to be determined whether cognitive impairment could have any relationship with other microvascular complications of DM, particularly DN. In line with this, we investigated whether the presence of DPN would be associated with cognitive functioning. Our most relevant findings were: i–patients with T2DM have a significant worse cognitive function than non-diabetic individuals; ii–no differences in cognitive function were found in T2DM patients with DPN in comparison to those without DPN and iii–no correlation was found between signs and/or symptoms of DPN and cognitive function.

Different mechanisms have already been proposed for DN pathogenesis. Some of these mechanisms have already been linked to the pathogenesis of cognitive impairment, including oxidative stress, microvascular vasculopathy, inflammation, dyslipidemia, among others [4–8]. Although these may be considered common mechanisms for both DN and cognitive impairment, no correlation between these two complications was found in our study. Moreover, since most of these variables were not evaluated in this study, it is not possible to determine whether any of these mechanisms could be related to DN and/or cognitive impairment in specific patients. Further studies are necessary to clarify this issue.

There are some explanations for our findings. First, the diagnosis of DPN was based only in clinical scales. Although these scales are widely used, it would be very interesting to compare findings from cognitive with nerve conduction tests. It should be noted that there were patients who scored less than 24 in MMSE, suggesting that individuals with dementia may have been included in the study. This might be of great relevance, particularly because NDS and NSS have not been validated in this specific population. NDS is based on the assessment of peripheral sensitivity, which completely depends on the ability of the patient to understand what is been asked and what is been done by the examiner. The same considerations can also be applied to NSS, which depends on the comprehension of specific questions to determine different symptoms. It is impossible to evaluate, at this moment, how cognitive impairment would influence the results of these scores (i.e. false-positives or false-negatives). However, it is worthy noticing that these scales have already been used in populations that included older individuals and dementia has not been evaluated in these studies (9,10). Second, the cognitive impairment that occurs in T2DM may have completely different mechanisms from the ones that affect peripheral nerves. Although these complications do share some similarities (as discussed above), multiple and individual components may play an important role in determining who will develop central and who will develop peripheral nerve destruction. Third, the vast majority of patients included in this study were women. Further studies are necessary to clarify whether the same results would also be demonstrated in men. Finally, there are other variables that could be of great relevance for the association of PDN and cognitive impairment, including Body Mass Index, Glycemic Control (including hypoglycemia), and lipid profile. Unfortunately, these variables were not accessed in this study.

In summary, cognitive impairment seems to be more severe in T2DM than in non-diabetic individuals. Although diabetic patients do have a worse cognitive function, this decline does not seem to be related to the presence and/or severity of DPN. Further studies are necessary to clarify whether these findings would also be applicable to different populations, with different stages of cognitive impairment and neuropathy.
